# Parenting Stress in Mothers of Children With Autism Without Intellectual Disability. Mediation of Behavioral Problems and Coping Strategies

**DOI:** 10.3389/fpsyg.2019.00464

**Published:** 2019-03-08

**Authors:** Ana Miranda, Alvaro Mira, Carmen Berenguer, Belen Rosello, Inmaculada Baixauli

**Affiliations:** ^1^Departamento de Psicología evolutiva y de la educación, Universitad de Valencia, Valencia, Spain; ^2^Departamento de Ciencias de la ocupación, logopedia, psicología evolutiva y de la educación, Universidad Católica de Valencia San Vicente Mártir, Valencia, Spain

**Keywords:** parental stress, social support, coping strategies, ASD, mediation analysis

## Abstract

The present study investigated the mediating role of behavioral difficulties, coping strategies, and social functional support in the relationship between symptoms severity and parenting stress in mothers of children with ASD (autism spectrum disorder). The parenting stress questionnaire, coping orientation to problems experienced scale, strengths and difficulties questionnaire, and Duke-UNC social support questionnaire were administered to 52 mothers, who also estimated the ASD severity symptoms of their children. Correlation analyses revealed that parenting stress was positively correlated with the children’s ASD symptoms and behavioral problems. On the other hand, parenting stress was negatively correlated with the engagement coping and social functional support reported by the mothers. Multiple mediation analysis indicated that engagement coping and behavioral difficulties were significant mediators in the relationship between ASD symptoms and parenting stress, with the engagement variable having a larger effect. The findings illustrate the need to promote the mothers’ engagement coping orientation and the application of behavioral strategies with their children to help them to buffer the impact of stress.

## Introduction

Autism spectrum disorder (ASD) is considered a complex neurodevelopmental disorder characterized by persistent alterations in communication and social interaction and by the presence of stereotyped patterns of behavior, activities, and interests (American Psychiatric Association, 2013). Its prevalence has multiplied by four in the past decade, with a rate of 1 in 59 children in the United States, according to the Center for Disease Control and Prevention (Baio et al., 2018). The data on the estimated prevalence in Spain, reaching 1.55% in preschoolers and 1.00% in school-age children, are close to the international ratings (Morales-Hidalgo et al., 2018).

Autism spectrum disorder is a lifetime, generally stable condition that involves persistent impairments in language, social skills, and daily life activities. Difficulties in child-rearing, which are present from early ages, put strong pressure on parenting skills. They can produce stress if the parents’ perceptions of the demands of their parental role exceed their coping resources, without being able to restore an equilibrium through the usual methods and strategies. The stress of parents of children with ASD, which reaches clinically significant levels in 77% of the cases (Kiami and Goodgold, 2017), is greater than the stress of parents with children with typical development (Davis and Carter, 2008; Hoffman et al., 2009; Rao and Beidel, 2009; Giovagnoli et al., 2015). Furthermore, it exceeds, with a large effect size, the stress of parents with children with other neurodevelopmental disorders, such as specific learning disorders, intellectual disabilities, Down syndrome, cerebral palsy, externalizing behaviors, or attention deficit hyperactivity disorder (Gupta, 2007; Hayes and Watson, 2013; Watson et al., 2013; Craig et al., 2016; Barroso et al., 2018).

During the decades of research on parenting stress, the construct has become more dynamic, integrating the capacity of the family system to successfully adapt to the significant challenge of raising a child with ASD. Scholars have shifted the focus from models based only on family crisis to those emphasizing adaptation. The Double ABCX Model of adjustment and adaptation (McCubbin and Patterson, 1983) emphasizes coping and social support as facilitators of the family’s adaptation to crisis, thus recognizing the importance of understanding influences that promote adaptation and buffer the effects of the risks. Parenting stress depends on a set of risk factors with interrelated strengths, including the personal characteristics of the individual with ASD, social support, and the family’s coping strategies. Together, these factors contribute to a family’s overall perceived stress and adaptation.

### Risk Factors of Parenting Stress

The conceptualization of autism as a spectrum disorder has resulted in a cohort of children with a wide range of behavioral profiles in terms of symptoms and presentations that may differentially impact parenting stress. At the cognitive or developmental level, symptom severity and the frequency and severity of behavioral difficulties are some of the relevant child characteristics that may act as stressors.

Although the greatest increase in the prevalence of ASD in recent years has occurred in the subgroup without intellectual handicap, referred to as “high-functioning autism” (Baio et al., 2018), the influence of the child’s IQ (intellectual quotient) level on family functioning has hardly been studied. Around 70% of parents of children with Asperger syndrome score at or above the 90th percentile of the normal parental stress scores (Mori et al., 2009). Therefore, it appears that a high level of cognitive development does not in itself buffer the effects of the stress produced by raising a child with ASD. Parents of children with high-functioning autism report significantly higher levels of stress and lower levels of adaptive coping and resources than parents of children with typical development. They also describe numerous negative experiences and lower quality of life, compared to parents of typically developing children (Lee et al., 2009).

In any case, it is necessary to examine the specific data found when analyzing different domains of parenting stress, in order to clarify the possible influence of cognitive development. The relationship that has arisen between higher stress levels and the difficult child from the PSI subcategory suggests that the parents of children with ASD without intellectual disability (ID) experience difficulties in managing the child’s basic behavior. This situation is related to the temperament of the child as well as learned patterns of defiance, non-compliance, and demanding behaviors (Bundy and Kunce, 2009; Mori et al., 2009). Along the same lines, the differences found have been on the total score on the PSI and the score in the child domain, but not in the parent domain. This means that the parents identify family stress as being directly related to behavioral factors of the child, such as hyperactivity, demandingness, and disturbed mood (Rao and Beidel, 2009). However, in other studies, the IQ level appears to be associated with parenting stress (Craig et al., 2016), specifically manipulative IQ (Pastor-Cerezuela et al., 2016).

Finally, behaviors that are specific to autism have shown the capacity to predict parental stress. The severity of the core features of autism is positively related to both parenting stress and maternal psychopathology symptoms (Tomeny, 2017). Specifically, self-isolated/ritualistic and repetitive behaviors are associated with poorer parent outcomes and with the mothers’ anxiety and depression, even when controlling for externalizing behaviors (Lecavalier et al., 2006; Bitsika and Sharpley, 2017). By contrast, other studies found no association between the severity of the autism symptoms and parental stress (Estes et al., 2009; Ben-Sasson et al., 2013; Giovagnoli et al., 2015).

Emotional and behavioral problems (EBPs) have been reported in children with ASD to a greater extent than what is commonly found in their TD peers (Bauminger et al., 2010; Strang et al., 2012). In a population sample, 92% of children with ASD had two or more comorbid problems (Posserud et al., 2018). The most common impairing comorbid conditions are attention-deficit/hyperactivity disorder, oppositional defiant disorder, peer problems, and anxiety disorder (Helland and Helland, 2017). The review by Barroso et al. (2018), based on 133 studies, documents the link between parental stress and the behavioral problems of children with ASD, especially highlighting externalizing problems (ES r weighted = 0.57, *d* = 1.39). The results agree with those from an exhaustive meta-analysis of the growing body of literature on the topic, which also concludes that relationships between additional EBP in children with ASD and psychological distress in their parents have been demonstrated (Yorke et al., 2018). These relationships are maintained over time, as the results of a longitudinal study with a follow-up assessment after 4 years reveal. Moreover, the lack of perceived change in the child’s problems is one of the factors that may justify, at least partly, the absence of changes in the levels of parenting stress, thus indicating the importance of intervention (Pozo and Sarriá, 2014b).

The relationship between EBP and parenting stress becomes more evident at around 6 years old (Zaidman-Zait et al., 2014). The parents probably focus their first concerns on “early autism” skill deficits, such as lack of joint attention, social responsiveness, or communication skills. However, when the children get older, parents may experience more stress because of internalizing and externalizing behaviors, which, in addition to being difficult to control, are socially stigmatizing.

Parenting stress is strongly predicted by EBP, above and beyond other characteristics of their children with autism, such as the severity of the autistic symptoms (Manning et al., 2011) and adaptive behaviors (Lecavalier et al., 2006). When investigating the relationship between EBP and parenting stress, interesting nuances emerge. The problems of children with ASD seem to affect different domains of parenting stress. Hence, behavioral and emotional problems significantly predict parental distress, whereas stress related to a dysfunctional parent–child relationship is associated with daily living, communication skills, and cognitive abilities (Giovagnoli et al., 2015).

### Protective Factors Against Parenting Stress

The characteristics of the child with ASD have been identified as a possible source of stress for families. However, some families, despite the difficulties, have still managed to achieve successful psychological adaptation. In particular, the analysis of the dynamics in families with chronic stressors requires complex models such as the Double ABCX Model of family stress and adaptation (McCubbin and Patterson, 1983). This model is able to discriminate between balanced and imbalanced families faced with chronic tension, obtaining a clearer picture of the differences.

The Double ABCX Model has been widely used to study the adjustment and adaptation process in families of children with disabilities, and specifically in families of children with ASD (Manning et al., 2011). In this model, family adaptation is considered a continuous variable ranging from maladaptation to bonadaptation. Maladaptation is defined as a continuous mismatch between the demands and the family’s capacity to cope with them, whereas bonadaptation is defined as a minimal discrepancy between the demands and the family’s capabilities, in order to achieve a balance in family functioning. Although it has undergone several revisions, the original formulation focusing on crisis variables in families is still relevant and in use: A (the crisis-precipitating event/stressor); B (the family’s crisis-meeting resources); C (the definition the family makes of the event), and X (the crisis). Thus, the model explains the factors associated with families’ adjustment to chronic stressors, including life tensions prior to or following the stressful event, the outcomes of family processes in response to the stressor (in terms of resources and the definition of the event), and finally factors that are part of the family’s attempt to adapt to the crisis, such as coping skills and social support.

Coping strategies refer to a group of behavioral or cognitive efforts aimed at reducing stress levels, and they are considered a tool parents can use to adapt to the stressors associated with raising a child with ASD. Coping strategies in studies with parents of children with ASD have generally been evaluated with the “Ways of Coping Questionnaire” (WOC; Folkman and Lazarus, 1988) or the “Coping Orientation to Problems Experienced” (COPE, Carver, 1997). Despite the different classifications proposed, two blocks of coping strategies have been emphasized: problem-focused coping strategies, which aim to solve the problem or do something to actively change one’s perception of a stressor (e.g., engagement and positive reframing), and emotion-focused coping strategies, which aim to reduce or manage the feelings of distress (e.g., escape-avoidance, denial, and distraction).

Parents who adopt positive and problem-focused strategies report less stress and better well-being than those who often use emotion-focused coping strategies, which are ineffective and do not resolve the adverse situation that provokes the stress (Dunn et al., 2001; Sivberg, 2002; Hastings et al., 2005a; Smith et al., 2008; Benson, 2010; Obeid and Daou, 2014; Lai et al., 2015; Kiami and Goodgold, 2017). Coping strategies and support needs serve as predictors of maternal stress, which declines by 0.402 points for each percent of increase in helpful coping strategies (Kiami and Goodgold, 2017).

However, child characteristics moderate the effect of coping strategies on maternal outcomes, so that engagement has a significant positive effect on maternal well-being in cases where the child’s symptoms are more severe (Benson, 2010). The mother’s tiredness has been revealed as an essential variable in this relationship. It is likely that the child’s behavioral difficulties contribute to maternal fatigue, which in turn may influence the use of ineffective coping strategies and increase stress. Thus, the unpredictable demands and changes in behavior create constant concern in the mothers about responding appropriately and restoring the balance, which produces a physical and mental fatigue that increases stress and the frequent application of ineffective strategies to deal with it (Seymour et al., 2013). The effects of the behavioral problems and the influence of coping strategies occur in parents of both sexes. Thus, for both fathers and mothers, the severity of the disorder and the behavioral problems have a direct and positive effect on stress, with the sense of coherence and active avoidance coping strategies playing a mediator role in both cases (Pozo and Sarriá, 2014a).

In any case, the comprehension of family adaptation processes when facing difficulties like those involved in raising a child with ASD is a complex issue, as a recent study pointed out. Through a latent profile analysis, four family profiles were identified based on socioeconomic risk, the use of coping strategies, family functioning, social support, and support focused on the family. In a follow-up evaluation 2 years later, the parents in the group characterized by Elevated Disengaged Coping and Limited Social Resources experienced the highest levels of stress and depression, and their children had significantly lower adaptive behavior scores and more behavioral problems than the children in the other three groups. The findings highlight the importance of disengagement and the low availability of social resources for the family in the social development of children with ASD (Zaidman-Zait et al., 2018).

Social support is defined as a multidimensional construct that includes physical and instrumental assistance, attitude transmission, resource and information sharing, and emotional and psychological support. The relationship between the scant social support for mothers of children with autism and stress has been supported in the literature. Low perceived social support, along with high repetitive behavior and low adaptive behavior, is a significant predictor of a greater perceived negative impact of having a child with ASD (Bishop et al., 2007). Studies have shown that more than 50% of mothers report a decreased ability to enjoy life as they face the challenges associated with raising a child with ASD. In fact, a low level of social support has been identified as the most powerful predictor of depression and anxiety in the mothers (Boyd, 2002). Conversely, social support plays an important buffering role against stress, especially for mothers (Pozo and Sarriá, 2014a), with a significant association found between receiving support from the family and social groups and the mothers’ ability to enjoy life (Al-Kandari et al., 2017). Higher levels of social support have been associated with lower levels of the negative impact generally produced by rearing a child with ASD, including psychological distress (Lindsey and Barry, 2018), negative mood, and depressive symptoms (Benson and Karlof, 2009).

Social support and self-efficacy are mediators of the family’s sense of control over life events and stressors (the hardiness) (Weiss et al., 2012). Likewise, parents who experience a higher level of social support also report a higher level of positive mood. Family support is associated with increased optimism, which predicts higher levels of positive maternal outcomes and lower levels of negative maternal outcomes (Ekas et al., 2010) and facilitates a better emotional relationship with the children (Boyd, 2002). Different sources of informal social support, including the partner, other family members, and friends, are factors that mediate and moderate maternal well-being, reduce stress, foster engagement (Sharabi and Marom-Golan, 2018), and predict changes in well-being, above and beyond the impact of the child’s behavioral problems (Manning et al., 2011; Smith et al., 2012).

In summary, the complexity of parenting stress in families of children with ASD requires a comprehensive approach in order to examine the possible influence of multiple variables simultaneously. Many researchers have investigated the predictors of stress in parents of school-aged children with autism, linking crucial factors such as symptom severity and the child’s behavioral problems, social support, and parents’ coping strategies. The age range and level of cognitive development of the individuals with ASD in the samples have been quite heterogeneous (Hastings et al., 2005a; Manning et al., 2011; Weiss et al., 2012; McStay et al., 2014; Pozo and Sarriá, 2014a,b; Zaidman-Zait et al., 2014, 2018; Giovagnoli et al., 2015). However, so far, few studies have investigated the predictors of parental stress in school children with ASD without intellectual disability, or “high functioning” (Bundy and Kunce, 2009; Lee et al., 2009; Mori et al., 2009; Craig et al., 2016). Moreover, we know of no research to date that has examined the relationship between ASD symptom severity and parenting stress using children’s behavioral problems, coping strategies, and social functional support as possible mediators in this relationship.

The current study is framed within the double ABCX model to examine the factors that influence parenting stress in a Spanish sample of families with a school-age child with autism without ID. The model is flexible and makes it possible to select which components to include. Its main strength is that rather than examining child characteristics, parent resources, and parental stress in an isolated way, it provides an overarching framework that includes each of these fundamental characteristics. In summary, this model enables us to analyze relationships among variables using multiple indicators and increase insight into the role of family resources that influence the family’s vulnerability to stress and its adaptation power.

From this perspective, the investigation had two objectives. The first was to analyze the relationships between parenting stress in families with ASD children without ID and the severity of the ASD symptoms, behavioral difficulties, coping strategies, and social support. The second objective was to investigate the possible mediator role of behavioral difficulties, coping strategies, and social support between ASD symptoms and parenting stress. We hypothesized that parenting stress would be positively correlated with ASD symptom severity, behavioral problems of the children, and coping strategies related to distraction and disengagement. In addition, parenting stress would be negatively correlated with engagement and cognitive reframing coping strategies and social confident and affective functional support. Finally, in a multiple mediation test, behavioral problems and positive coping strategies and social support were expected to emerge as significant mediators on the path between ASD symptom severity and parenting stress.

## Materials and Methods

### Participants

Participants in this study were 52 families with children with an autism diagnosis. All the families lived in the Valencian community and were recruited through public schools and parental support groups. A member of the team made initial telephone contact with the mothers to explain the objectives of the study and request their collaboration and that of their children.

Most of the children were boys (48), and ages ranged from 7 to 11, with a mean age of 8.59 (*SD* = 1.38). They had received a clinical diagnosis of an autism spectrum condition in the Psychiatry and Child Neurology services of hospitals and medical centers in the Valencian community at ages ranging between 2 years and 11 months and 6 years (mean age of diagnosis = 4.69; *SD* = 1.67). The diagnosis was generally made using a multi-team approach, based on the Diagnostic and Statistical Manual of Mental Disorders (DSM-5; American Psychiatric Association, 2013), the Autistic Diagnostic Interview—Revised (ADI-R; Rutter et al., 2006), and/or Autism Diagnostic Observation Schedule-WPS (ADOS-WPS; Lord et al., 2000). Children diagnosed with Asperger Syndrome comprised 31 participants of the study, and the remaining diagnoses included Autism Spectrum Disorder (11 children) and Pervasive Developmental Disorder (10 children). In order to confirm the ASD diagnosis years later for the present study, strict cutoff scores were used, as recommended on the Social Communication Questionnaire (SCQ; Rutter et al., 2003) and on the ADI-R (Rutter et al., 2006) and diagnostic criteria for ASD from the fifth edition of the DSM-5 (American Psychiatric Association, 2013), specifically the three criteria from block A on socio-communicative impairments and at least two criteria from block B on repetitive behaviors and restricted interests. Thus, this procedure verified that the children who were evaluated, except two cases, met the cutoff scores required for the diagnosis.

All the children had an intelligence equal to or above 80, measured on the K-BIT intelligence test (Kaufman and Kaufman, 2000), which comprises subtests measuring verbal and non-verbal intelligence. Language development, evaluated with the vocabulary subtest from the WISC-IV (Wechsler, 2003), which is closely related to overall language, also fell within the average range, with a mean of 11.51 (*SD* = 3.34). The children attended classes in ordinary schools, although they were receiving extra educational support of varying degrees (20 children were enrolled in communication and language classrooms).

In terms of mother and family characteristics, the mothers’ mean age was 40.17 (*SD* = 4.82). With regard to their education level, 29 had obtained a university degree, 6 had obtained a high school diploma, and 17 had studies corresponding to primary or secondary education. A majority of mothers were employed (33), whereas 9 were unemployed, and 10 exclusively fulfilled the role of housewife. The majority of the mothers were married (78.84%), and the rest were separated/ divorced (19.3%) or single (1.9%).

Demographic data are summarized in Table 1.

**Table 1:** Sociodemographic characteristics of the sample (*N* = 52).

Children demographics	M (SD)	Mothers’ demographics	M (SD)
Age	8.59 (1.38)	Age	40.17 (4.82)
IQ	101.42 (12.65)	Mother’s education	2.94 (1.33)
Vocabulary	11.51 (3.34)	Primary education N (%)	15 (28.84)
Number of siblings	0.84 (0.65)	Secondary education N (%)	2 (3.84)
Sex (boys) N (%)	48 (92.3)	High school N (%)	6 (11.53)
Medication N (%)	17 (32.7)	University degree N (%)	29 (55.76)
Repeated courses N (%)	3 (5.76)	Employment status	0.71 (1.12)
Educational support N (%)	52 (100.0)	Employed N (%)	33 (63.46)
ADI-R A	13.49 (2.94)	Unemployed N (%)	19 (36.53)
ADI-R B	8.91 (2.55)	Marital status	0.40 (0.79)
ADI-R C	4.70 (2.03)	Married N (%)	41 (78.84)
SCQ	22.51 (7.01)	Other N (%)	11 (21.15)


*M, mean; SD, standard deviation; ADI-R, autism diagnostic interview-revised; ADI-R A, qualitative alterations in the reciprocal social interaction; ADI-R B, qualitative alterations in communication; ADI-R C, restrictive and stereotyped behaviors; SCQ, Social Communication Questionnaire.*

### Measures

#### Family Demographic Information

In the first interview held with the mothers individually, the family’s most relevant sociodemographic data were collected: demographics (i.e., age, gender, marital status), socio-economic status (i.e., profession, education level, job situation), and family structure variables (i.e., number of children in the family), as well as the child’s characteristics (i.e., age, gender, diagnosis, age when diagnosed, medication, academic performance, support in school).

#### Measurement of Stress

##### Parenting Stress Index – Short Form (PSI-SF; Abidin, 1995; adapted to Spanish by Díaz-Herrero et al., 2010)

This scale is a self-report measure filled out by the parents. It contains 36 items distributed in three subscales of 12 items each, rated on a five-point Likert-type response scale. The first scale, parental distress, evaluates the distress experienced by parents due to personal factors, such as depression or conflict with a partner, or life restrictions due to the demands of childrearing in their role as parents (i.e., “Since having my child, I feel that I am almost never able to do things I like to do.” The second scale, parent–child dysfunctional interaction, provides information about the parents’ feelings about the interactions with their child and the degree of frustration of the expectations and trust they have placed in their child (i.e., “Most times, I feel that my child does not like me and does not want to be close to me”). The third scale, difficult child, is designed to measure the parents’ perceptions of their child’s self-regulatory abilities (i.e., “My child seems to cry or fuss more often than most children”). The scale also provides a measure of total stress by adding up the scores on the 36 items, with a total score above 90 being clinically significant.

The Cronbach’s alpha internal consistency coefficients in our sample were: parental distress (0.91), dysfunctional parent–child interaction (0.82), and difficult child (0.90), which are similar to those obtained in other studies carried out in Spain (Diaz-Herrero et al., 2011). It is the most widely used instrument to evaluate stress in studies on ASD; in fact, it was utilized in 75% of the studies included in a recent systematic review (Barroso et al., 2018).

#### Coping Measure

##### Coping Orientation to Problems Experienced (Brief COPE- Carver, 1997; Spanish adaptation by Morán et al., 2010)

This scale focuses on evaluating the coping strategies people use when facing stress. It has 14 subscales and 28 items that are responded to on a four-point Likert-type scale (from 0 to 3), ranging from “I never do this” to “I always do this,” with intermediate scores. In this study, the Benson (2010) factorization was used, which groups the coping strategies in four main factors: Factor 1 (engagement), which corresponds closely to the category of problem-focused coping as conceptualized in the stress literature (Lazarus and Folkman, 1984) and includes four subscales (use of instrumental support, active coping, planning, and use of emotional support), all of which reflect the mother’s active involvement in addressing the stressful situation posed by her child’s autism. Factor 2 (distraction) also includes four subscales (self-distraction, humor, self-blame, and venting) reflecting the mother’s efforts to cope with her child’s autism by letting off steam and modulating emotion. Factor 3 (disengagement) includes three subscales (substance use, behavioral disengagement, and denial) involving attempts by the mother to deny or distance herself from the situation. Finally, Factor 4 (cognitive reframing) includes three subscales (acceptance, use of religion, and positive reframing), all of which describe the mother’s efforts to cope with her child’s autism in a positive way through acceptance, cognitive restructuring, and use of religion.

Cronbach’s alpha reliabilities indicate good internal consistency for each of the four coping dimensions, with values ranging from 0.73 (distraction) to 0.86 (engagement) (Benson, 2010). In our sample, the Cronbach’s α range between 0.71 (disengagement) and 0.77 (engagement), values similar to those obtained with the Spanish version: 0.74 to 0.80 (Morán et al., 2010).

#### Social Support Measure

##### Social Functional Support Questionnaire Duke-UNC (Broadhead et al., 1988; adaptation to the Spanish population by Bellón Saameño et al., 1996)

This questionnaire is filled out by parents and rates perceived support through two scales with a total of 11 items, using a five-point Likert-type response scale (from1 point “much less than what I would like” to 5 points “as much as I like”). The confident social support scale has a total of six items that rate the possibility of having people to whom you communicate intimate thoughts (“I have the possibility of talking to someone about my problems at work or at home”). The other scale, affective social support, includes five items designed to evaluate demonstrations of caring and empathy (“I get help with things related to my house”). In this study, the direct scores on both scales were used. Consistent with the results of the Spanish adaptation (Bellón Saameño et al., 1996), internal consistency (Cronbacht’s α) of the confidential support and affective scales for our sample was 0.83 and 0.74, respectively.

#### Child Behavioral Problems Measure

##### Strengths and difficulties questionnaire (SDQ-Cas; Goodman, 1997; adapted to the Spanish population by Rodríguez-Hernández et al., 2012)

The SDQ can be filled out by parents or teachers to rate a wide range of psychopathological symptoms and prosocial behavior in children and adolescents between 4 and 16 years old. It has five scales that rate emotional symptoms (“Often unhappy, depressed or tearful”), behavioral problems (“often fights with other children or bullies them”), relationship problems with peers (“Rather solitary, prefers to play alone”), hyperactivity (“Restless, overactive, cannot stay still for long”), and prosocial behavior (“Considerate of other people’s feelings”). Each of the five scales incorporates 3 response alternatives (not true, somewhat true, very true). In addition, a total score for difficulties can be extracted by adding up the scores on the first four scales. The score on the total difficulties scale can range from 0 to 40 points, with the Spanish version establishing a cutoff for “abnormal” scores at values between 20 and 40 points. The SDQ presents adequate psychometric properties with adequate reliability (0.73) (Goodman, 2001), confirmed in the Spanish population (0.76) (Rodríguez-Hernández et al., 2012). In our study, the questionnaire was administered to the parents, and the total score for difficulties was used for the analyses, with a Cronbach’s α = 0.83 for our sample.

#### Child Autism Symptoms

##### ASD clinical criteria from the DSM-V (American Psychiatric Association, 2013)

The severity of the ASD symptoms was assessed through an interview between the parents and a clinical psychologist, focused on the seven diagnostic criteria for the disorder. The first three were to evaluate socio-communicative impairments, and the other four to rate repetitive behaviors and restricted interests. Through the interviews, the parents evaluated the severity of each criterion using a 4-point Likert scale ranging from 0 to 3, where 0 represents “almost never,” 1 “sometimes,” 2 “often,” and 3 “many times.” Therefore, a higher score on the DSM-V indicates greater severity of the ASD symptoms.

### Procedure

This study obtained the approval of the University of Valencia ethics committee for research with humans (Declaration of Helsinki in the Convention of the European Council, 1964). Likewise, the Department of Education of the Valencian Government gave permission to locate the study participants who had previously received a diagnosis of ASD by professionals specialized in children’s public health. After being informed about the objectives, the parents signed the consent forms to participate in the study, and they were fully aware that they could drop out if they so desired. Next, an individual interview session was held with the parents to complete the demographic data, and the ADI-R interview was administered by an accredited psychologist to confirm the diagnosis. In addition, in a later session, one of the members of the team filled out the PSI, SDQ, COPE, and social support questionnaires with the mother, in order to extract the necessary data on their children to carry out this study.

### Data Analyses

The statistical analyses were performed with the statistical program for the Social Sciences (SPSS), version 24. Preliminary analyses were conducted to examine the distribution of the variables using the Kolmogorov–Smirnov test; variables that did not show a normal distribution were transformed using square-root transformation.

To examine the relationship between the ASD Symptoms, PSI, SDQ, coping skills, and social support in families with children with ASD, Pearson correlations were calculated. A multiple regression analysis was also carried out to study the impact of the variables on the parenting stress index. Finally, a multiple mediation analysis was performed using the PROCESS program of mediation, moderation, and conditional analysis (Hayes, 2013), in order to examine the possible mediation of the variables that showed statistical significance with parenting stress: SDQ total difficulties and engagement. This approach uses boot-strapping to estimate the parameters, which is particularly appropriate for small samples. The ASD Symptoms variable was used as the independent variable, the parenting stress index was the dependent variable, and SDQ and engagement were used as mediator variables. In the mediation analyses, the bootstrap procedure with 10,000 repetitions was used to verify the mediator effect of the aforementioned variables, with a confidence interval of 95%. When the indirect effect of the mediators is significantly different from zero, this indirect effect is considered significant.

**FIGURE 1:**
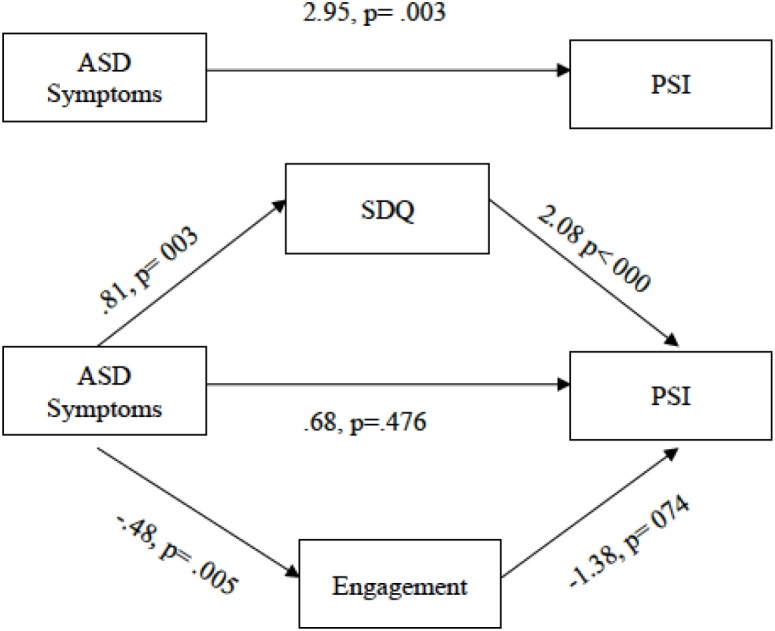
Multiple mediation analyses of direct effect of the ASD symptoms on the parenting stress index for the two mediators (SDQ and Engagement).

According to Baron and Kenny (1986), in order for the model to be acceptable, four assumptions must be met: (a) the predictor variable must be related to the dependent variable; (b) the predictor variable must be related to the mediator; and (c) the mediator variable must be related to the dependent variable after controlling the effect of the predictor variable. The final condition is that the effect of the predictor variable on the dependent variable, controlling the effect of the mediator variable, must not be statistically significant. In the present study, due to the sample size, we followed the recommendations of MacKinnon et al. (2004), designed specifically to obtain reliable and valid conclusions in studies where the sample samples are not large (Preacher and Hayes, 2004). These pathways are shown in Figure 1.

## Results

### Relationships Among ASD Symptoms, PSI, SDQ, Coping Skills, and Social Support in Families With Children With ASD

Bivariate correlations between the study variables and parenting stress are shown in Table 2.

**Table 2:** Pearson correlations between ASD symptoms, PSI, SDQ, coping skills, and social support.

	M (SD)	1	2	3	4	5	6	7	8	9
(1) ASD Symptoms	16.00 (3.13)	-								
(2) PSI_Tot	93.45 (23.04)	0.40**	-							
(3) SDQ_Tot	19.63 (6.23)	0.39**	0.61**	-						
(4) Engagement	16.80 (3.55)	-0.36**	-0.28*	-0.05	-					
(5) Distraction	7.78 (4.48)	0.16	0.16	0.17	0.03	-				
(6) Disengagement	1.21 (1.95)	0.21	0.16	0.03	-0.19	0.41**	-			
(7) Cognitive Re.	10.28 (3.10)	-0.25	-0.25	-0.18	0.23	0.03	-0.15	-		
(8) Confidant S.	21.16 (5.47)	-00.45**	-0.28*	-0.31*	0.48**	0.13	-0.09	0.01	-	
(9) Affective S.	17.54 (3.69)	-0.28*	-0.09	-0.14	0.33*	0.15	0.02	0.02	0.70**	-


*ASD Symptoms, autism symptoms; PSI_Tot, Parenting Stress Index total score; SDQ Tot, Strength and Difficulties Questionnaire total score; Cognitive Re., cognitive reframing; S., support. ^∗^*p* < 0.05, ^∗∗^*p* < 0.01.*

Pearson product-moment correlation analyses revealed the existence of significant associations between the ASD symptoms from the DSM 5 and the parenting stress total index (*p* < 0.001), SDQ (*p* < 0.001), engagement (*p* < 0.001), confidant support (*p* < 0.001), and affective support (*p* < 0.05). In addition, the analyses revealed significant correlations between the parenting stress total index and the SDQ (*p* < 0.001), engagement (*p* = 0.04), and confidant support (*p* = 0.05).

Multiple regression analysis was carried out in the ASD group in order to evaluate the relationships between the dependent variable of parenting stress and the ASD symptoms, SDQ, engagement, and confidant support as explanatory variables. The results of the regression analysis indicated that the significant predictors that explained the highest percentage of variance in parenting stress were the SDQ (β = 0.56, *p* < 0.001) and engagement (β = -0.26, *p* = 0.05). However, confidant support was not a significant predictor of parenting stress (β = 0.06, *p* = 0.63) (Table 3). It is possible that the communality with the contents evaluated by the instrumental support subscale of engagement would explain the fact that the confident support scale from the Duke-UNC questionnaire did not reach the level of statistical significance in the regression equation.

**Table 3:** Multiple regression analysis of ASD symptoms, SDQ, engagement, and confidant support predicting parenting stress in the ASD group.

	*B*	*SE*	β	*p*
PSI_Tot	*F*(4,44) = 8.60^∗^; *R^2^* = 0.39			
ASD Symptoms	0.60	1.0	0.09	0.52
SDQ_Tot	2.08	0.46	0.56	0.00^∗∗^
Engagement	-1.7	0.87	-0.26	0.05*
Confidant S.	0.28	0.58	0.06	0.63


*ASD Symptoms, autism symptoms; PSI_Tot, Parenting Stress Index total score; SDQ_Tot, Strength and Difficulties Questionnaire total score; Confidant S., confidant support. ^∗^*p* < 0.05, ^∗∗^*p* < 0.01.*

The results of the correlations and regression analyses supported the viability of the mediation because the four assumptions Baron and Kenny (1986) were met.

### Multiple Mediation Analyses Between ASD Symptoms and the Parenting Stress Index, Controlling for SDQ and Engagement

To examine explanatory mechanisms underlying the significant predictors of PSI and ASD symptoms described above, we tested the role of engagement skills and behavioral and emotional problems as mediators. Multiple mediation analysis, which simultaneously examines multiple indirect effects, potentially extends the comprehension of the causality phenomenon. This analysis identifies indirect effects by controlling for all the other mediators, and it reduces the alpha inflation and investigates several indirect effects simultaneously, rather than in a series of single mediator models (Preacher and Hayes, 2008).

Figure 1 shows a multiple mediation model in which the SDQ and engagement mediate the relationship between ASD symptoms and the parenting stress index. As Figure 1 shows, when the direct effect of ASD symptoms on the parenting stress index was evaluated, controlling for the mediator variables, path c’ was not statistically significant (*p* = 0.48). In addition, the ASD symptoms had a significant direct effect on path α1, CI [0.28, 1.34] and path α2, CI [-0.82, -0.15]. The total effect (c) of the relationship between ASD symptoms and Parenting stress was statistically significant (*p* = 0.003).

In the next step, the standardized indirect effects in the relationship between ASD symptoms and PSI were calculated to test the possible statistical significance of the two mediators introduced in the model, following the suggestions by Hayes and Rockwood (2017). The bootstrapping procedure was used for a sample of 10,000 and a confidence interval (CI) of 95% (Table 4). The results showed that, controlling for the SDQ variable, the value of 0 was not included in the confidence interval, indicating that there was a significant standardized indirect effect, CI [-0.04, 0.44]. Similarly, when the engagement variable was controlled, the results showed a significant standardized indirect effect, CI [0.01, 0.22].

**Table 4:** Completely standardized indirect effects and contrasts between SDQ and engagement indirect effects on the path from ASD symptoms to PSI mediated b.

	*Effect*	*SE*	BootLLCI	BootULCI
**Indirect effects**	
SDQ	0.23	0.10	0.042	0.444
Engagement	0.09	0.05	0.008	0.216
SDQ, Engagement	0.01	0.02	-0.048	0.020
Total indirect effect	0.31	0.11	0.109	0.540
**Contrasts**				
SDQ vs. Engagement	0.14	0.12	-0.082	0.377
Engagement vs. SDQ	0.24	0.10	0.043	0.467


Finally, an examination of the pairwise contrast of the indirect effects showed that the engagement variable had a larger effect, CI [0.043, 0.467] than the SDQ, CI [-0.082, 0.377], as Table 4 shows.

## Discussion

The present study is framed in the line of research focused on the double ABCX model (McCubbin and Patterson, 1983). Its principal aim was to integrate characteristics of the child and characteristics of the parents in the analysis of parenting stress in families with a school-age child with autism without ID. In this subgroup, parenting stress and its relationship with risk or protector factors have hardly been studied, in spite of the increase in its prevalence in the past decade (Baio et al., 2018).

The first objective was to identify the relationships among different variables under study, specifically parenting stress and severity of ASD symptoms, behavioral difficulties, coping strategies, and social support. The significant association values found between the different variables were generally in the expected direction. ASD symptoms were positively related to parenting stress, indicating that the increase in the core symptomatology of ASD was related to a sharp increase in maternal distress. The data coincide with previous findings (Lecavalier et al., 2006; Bitsika and Sharpley, 2017; Tomeny, 2017), although in other studies, no association was found between the severity of the autism symptoms and parental stress (Estes et al., 2009; Ben-Sasson et al., 2013; Giovagnoli et al., 2015). Likewise, the positive relationship between ASD symptoms and behavioral problems was confirmed, as consistently reported in the literature (Helland and Helland, 2017; Posserud et al., 2018). The ASD symptoms, as expected, were significantly and negatively associated with engagement coping and with the two functional social support scales, confidant support and affective support. This result suggests that mothers who perceive the autism symptoms of their children with greater intensity tend to reduce their problem-focused coping strategies, think they can communicate their intimate feelings to other people less, and receive fewer demonstrations of caring and empathy.

Regarding parenting stress, the correlations generally support our hypotheses about the expected relationships. In addition to being correlated with ASD symptoms, parenting stress presented a positive association with behavioral problems, exceeding the value for the relationship between ASD symptoms and behavioral problems. The results agree with the conclusions of systematic reviews and meta-analyses supporting the important link between parenting stress and EBPs of children with ASD (Barroso et al., 2018; Yorke et al., 2018). Moreover, parenting stress showed a significant negative relationship with engagement and confidant support. Therefore, the findings reiterate that positive and problem-focused strategies are associated with less parenting stress (Dunn et al., 2001; Sivberg, 2002; Hastings et al., 2005a; Smith et al., 2008; Benson, 2010; Obeid and Daou, 2014; Lai et al., 2015; Kiami and Goodgold, 2017), and that social support can also have a buffering effect on the psychological distress produced by raising a child with ASD (Lindsey and Barry, 2018), especially for the mothers (Pozo and Sarriá, 2014a). Other negative coping strategies, such as distraction and disengagement, did not reach the level of statistical significance of 0.05 in the relationship with parenting stress, thus playing a much less important role. By contrast, other studies have found that the use of maladaptive coping behaviors was associated with high levels of maternal stress (Hastings, 2002; Seymour et al., 2013). This incongruence may be due to the fact that the sensitivity of parental coping strategies to Hispanic cultural influences leads to underreporting negative coping strategies that involve the mother’s attempts to deny or distance herself from the situation.

However, the analysis of parenting stress must be considered from a more complex perspective than simple bivariate relationships. These relationships may be modified by a third variable, such as mediator (Baron and Kenny, 1986), which could provide information about how or why two variables are related. In fact, another objective of the present study was to investigate the possible mediator role of behavioral difficulties, engagement, and social support in the link between ASD symptoms and parenting stress. In the previous multiple regression analysis carried out to evaluate the relationships between the dependent variable of parenting stress and these three explanatory variables, confident support (Duke-UNC) did not reach the level of statistical significance in the regression equation, probably because it overlaps with contents that are evaluated more intensively by engagement coping.

Due to the possibility that various mediators could be involved in the relationship between ASD symptoms and parenting stress, it is considered more accurate and parsimonious to include all of them in the same model in order to simultaneously investigate various indirect effects, rather than using a series of single-mediator models (Preacher and Hayes, 2008). The results of the multiple mediation model showed that both the behavioral problems (SDQ) and engagement coping mediated the relationship between ASD symptoms and parenting stress. It is evident that the severity of the behavioral problems of the child with ASD, independently of his/her cognitive development, requires extraordinary resources that impede the appropriate response to difficult situations that arise daily, producing stress in the mothers (Bauminger et al., 2010). Engagement, which includes the use of instrumental support, active coping, planning, and emotional support, all of which reflect the mother’s active involvement in addressing the stressful situation, significantly mediated maternal stress in dealing with the autism symptoms. This tendency is consistent with what was found in other studies in which higher levels of problem-focused coping were generally associated with greater well-being, acting as a buffer when autism symptoms were high (Smith et al., 2008).

Finally, examining the pairwise contrast of the indirect effects, the engagement variable had a larger effect than the child’s behavioral problems. This result supports the magnitude of the role played by positive coping strategies as protector factors against maternal stress. Moreover, its crucial influence can be felt over time. An exceptional longitudinal study showed that the persistent stress experienced by mothers of children with ASD is exacerbated when personal and social resources are insufficient. Mothers who utilized more active coping strategies and relied less on disengaged coping strategies, either at the time of diagnosis or over time, experienced lower levels of parenting stress (Zaidman-Zait et al., 2017).

### Limitations and Future Directions

To the best of our knowledge the present study is the first one to explore parenting stress in parents of children with ASD without ID and the potential mediating effects of behavioral problems and coping strategies. Nonetheless, it has various limitations. The sample included a small number of participants, and so the results have to be considered preliminary, and replication studies are needed. Furthermore, the results only apply to children with ASD who have an IQ within normal limits, which may also restrict generalizability. Using a group with ASD and ID would have allowed us to draw firmer and more generalizable conclusions. However, at the same time, the homogeneity of the sample’s intellectual characteristics and age is a strength due to its specificity. Additionally, most of the children were male, and so the results might not be generalizable to girls with ASD. Information was obtained through parent-report questionnaires. These tests are well-validated, but future work should use multi-informant assessments to obtain more ecological measures of the predictor variables.

The informants were mothers because both parents attended the evaluation sessions in only four cases. However, mothers are more often the primary caregivers of children in Spanish culture, and other studies found no differences in the stress levels of fathers and mothers of children with ASD (Hastings et al., 2005b; Davis and Carter, 2008; Stanojević et al., 2017). Another potential problem was that the same mothers who rated their child’s EBP evaluated their own stress and coping strategies; consequently, correlations could be inflated by common method effects. In our study, questionnaire data collected from the same source may show relationships for reasons other than a real association between the constructs of interest. Another doubt that arises is whether the aspects of the child’s behavior that the parents consider problematic would coincide with the rating of other significant people, such as the teachers. However, in any case, parent agreement with other informants appears to be moderate for the externalizing and internalizing behavioral problems of children with ASD (Stratis and Lecavalier, 2015), indicating that their perception shares some common variance with that of other informants.

Our study analyses did not consider economic factors, such as income, or the number of family members, which are important variables in evaluating parenting stress. Some data indicate that groups of parents of children with ASD with a greater socioeconomic disadvantage show higher stress and depression levels (Stanojević et al., 2017; Zaidman-Zait et al., 2018). A positive point is that our sample was fairly homogeneous on two factors. Most of the mothers had high school level studies or a university degree and one or two children.

### Implications

Along with the limitations, the practical aspects of the main findings of this study should be emphasized. A normalized intelligence level of children with autism is not a factor protecting mothers from stress. The mean score on parenting stress of the mothers in our sample exceeded the cutoff score for clinical significance. These high levels of maternal stress also remain stable throughout the key stages from preschool to early high school (McStay et al., 2014), thus impacting parenting behavior.

Eliminating the stressors mothers face in raising children with ASD does not seem possible. Instead, improving parental coping and resilience should be the objective in helping family functioning when there are new and ongoing challenges. Parents must receive help through family-centered supportive services that offer counseling, in order to decrease their stress levels by using appropriate coping strategies and other resources. Brief interventions that include stress management, details about specific behavioral impairments, and principles of behavior management within a set of components (information on autism, strategies for teaching new skills, improving social interaction and communication, service availability, family and community responses to autism) have shown their effectiveness in reducing parenting stress and improving family life (Kasari et al., 2015). Furthermore, according to an emerging body of research, mindfulness-based interventions may be helpful in reducing parenting stress in mothers who have children with ASD (Conner and White, 2014).

## Data Availability

The datasets generated for this study are available on request to the corresponding author.

## Author Contributions

AnM and AlM designed the research. CB and BR performed the evaluations. CB analyzed the data. AnM, BR, and IB contributed to the final wriring of the manuscript. All authors listed revised the manuscript.

## Conflict of Interest Statement

The authors declare that the research was conducted in the absence of any commercial or financial relationships that could be construed as a potential conflict of interest.
